# Distribution of Heparan Sulfate Oligosaccharides in Murine Mucopolysaccharidosis Type IIIA

**DOI:** 10.3390/metabo4041088

**Published:** 2014-12-11

**Authors:** Kerryn Mason, Peter Meikle, John Hopwood, Maria Fuller

**Affiliations:** 1 Forensic Science South Australia, 21 Divett Place, Adelaide, South Australia 5000, Australia; E-Mail: kerryn.mason@sa.gov.au; 2 Baker ID Heart and Diabetes Institute, 75 Commercial Road, Melbourne, Victoria 3006, Australia; E-Mail: peter.meikle@bakeridi.edu.au; 3 SAHMRI, North Terrace, Adelaide, South Australia, 5000 Australia; E-Mail: john.hopwood@sahmri.com; 4 SA Pathology at Women’s and Children’s Hospital, 72 King William Road, North Adelaide 5006, Australia; E-Mail: maria.fuller@adelaide.edu.au

**Keywords:** lysosomal storage disorder, mouse model, mucopolysaccharidosis type IIIA, heparan sulfate, oligosaccharides, reverse phase high performance liquid chromatography, electrospray ionization-tandem mass spectrometry

## Abstract

Heparan sulfate (HS) catabolism begins with endo-degradation of the polysaccharide to smaller HS oligosaccharides, followed by the sequential action of exo-enzymes to reduce these oligosaccharides to monosaccharides and inorganic sulfate. In mucopolysaccharidosis type IIIA (MPS IIIA) the exo-enzyme, N-sulfoglucosamine sulfohydrolase, is deficient resulting in an inability to hydrolyze non-reducing end glucosamine N-sulfate esters. Consequently, partially degraded HS oligosaccharides with non-reducing end glucosamine sulfate esters accumulate. We investigated the distribution of these HS oligosaccharides in tissues of a mouse model of MPS IIIA using high performance liquid chromatography electrospray ionization-tandem mass spectrometry. Oligosaccharide levels were compared to total uronic acid (UA), which was used as a measure of total glycosaminoglycan. Ten oligosaccharides, ranging in size from di- to hexasaccharides, were present in all the tissues examined including brain, spleen, lung, heart, liver, kidney and urine. However, the relative levels varied up to 10-fold, suggesting different levels of HS turnover and storage. The relationship between the di- and tetrasaccharides and total UA was tissue specific with spleen and kidney showing a different disaccharide:total UA ratio than the other tissues. The hexasaccharides showed a stronger correlation with total UA in all tissue types suggesting that hexasaccharides may more accurately reflect the storage burden in these tissues.

## 1. Introduction

Degradation of the glycosaminoglycan (GAG), HS, begins with endo-degradation of the polysaccharide to smaller HS fragments, followed by the sequential action of lysosomal exo-enzymes to reduce these oligosaccharides to monosaccharides and inorganic sulfate for reutilization by the cell. In an inherited metabolic disorder known as MPS IIIA the lysosomal exo-enzyme, N-sulfoglucosamine sulfohydrolase (SGSH; EC 3.10.1.1), responsible for cleaving sulfate from non-reducing end glucosamine N-sulfate (GlcNS) residues in HS is deficient. Consequently, partially degraded HS oligosaccharides accumulate in the lysosomes of SGSH deficient cells, resulting in their urinary secretion, and chronic and progressive deterioration of cells, tissues and organs [1].

Total urinary GAG is often used as a biochemical measure of disease activity in MPS IIIA using cetylpyrinium chloride and ethanol precipitation of GAG followed by the measurement of free and conjugated UA by the hydroxydiphenol method [2]. This has particular application for assessing the biochemical response to therapies in the MPS IIIA mouse model. Roberts *et al*. [3] reported that total urinary GAG was decreased in MPS IIIA mice after treatment with rhodamine B, and using the same procedure for precipitating GAG, followed by gradient polyacrylamide gel electrophoresis, total urinary GAG decreased in MPS IIIA mice treated with a lentiviral-mediated gene correction vector [4]. In another study, to provide for a non-subjective analysis of lysosomal storage in MPS IIIA mouse tissues, the total UA present in liver, heart, kidney, spleen and brain was determined in tissue macerates using the hydroxydiphenyl method: total UA was significantly elevated in all MPS IIIA tissues tested compared to normal controls [5]. While changes in GAG and UA have been reported in MPS IIIA mouse tissues and urine, these methods measure a mixture of oligo- and polysaccharides and do not discriminate between oligosaccharides derived from HS, chondroitin sulfate or dermatan sulfate.

The development of sensitive mass spectrometry based technology has facilitated the measurement of partially degraded HS oligosaccharides in complex biological samples such as cultured fibroblasts, blood and urine [6,7,8,9,10,11]. In contrast to the measurement of total GAG, which represents a mixture of SGSH-substrate and non-substrate oligo- and polysaccharides, these oligosaccharides represent the actual SGSH- substrate, containing a terminal GlcNS residue, and so have the potential to provide a more accurate measure of HS substrate burden. HS in serum and plasma has also been digested with heparitinase producing a series of HS disaccharides which, when analyzed by HPLC electrospray ionization-tandem mass spectrometry (ESI-MS/MS) were shown to be elevated in MPS IIIA patients when compared to levels in control groups [12,13]. However, the complete digestion of HS to disaccharides with bacterial endo-enzymes prior to HPLC ESI-MS/MS prohibits the investigation of the structural composition of the partially degraded HS oligosaccharides in MPS IIIA.

We have previously proposed the use of a HS-derived disaccharide to biochemically monitor the effects of therapy for MPS IIIA [14,15,16,17,18,19]. Using ESI-MS/MS in combination with enzyme and chemical digestion we also identified mono- to hexadecasaccharides in the urine of an MPS IIIA patient [20]. These oligosaccharides were composed of N-acetylated (GlcNAc-UA) and unsubstituted glucosamine (GlcN-UA) repeating disaccharides with up to two sulfates per disaccharide. The glucosamine-uronic acid disaccharide with one sulfate group (GlcN-UA (+1S), GlcNS-UA) has previously been shown to be elevated in a cohort of human MPS IIIA urines compared to unaffected controls [7].

Despite the use of partially degraded HS oligosaccharides and total UA as measures of storage burden in MPS IIIA, the relationship between them remains largely unknown. Here we have introduced HPLC separation prior to MS analysis to enable measurement of di- to hexasaccharides and permit their correlation with total UA in tissues and urine of the MPS IIIA mouse model. We also assessed whether the oligosaccharides detected in the urine reflect a contribution of the oligosaccharides in all tissues or just those present in the kidney.

## 2. Results

### 2.1. UA in Mouse Tissues and Urine

**Figure 1:**
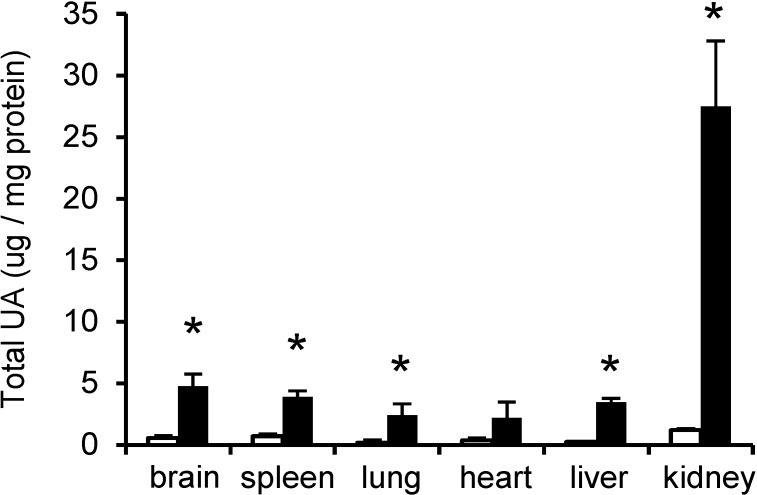
Total UA in wild type and MPS IIIA mouse tissues. GAG was isolated from the tissues of 30-week-old MPS IIIA mice (*n* = 3) and age-matched wild type mice (*n* = 3) using anion exchange chromatography. Eluates were assayed for UA and results expressed as µg UA/mg of total protein. Data points represent the mean plus one standard deviation for wild type (open bars) and MPS IIIA (filled bars) mice. *p ≤ 0.05 compared to wild type mice.

A similar distribution of UA was seen in the wild type and MPS IIIA mouse tissues with the kidney showing the highest level (Figure 1). Total UA was significantly elevated (*p* < 0.05) in the MPS IIIA brain, spleen, lung, liver and kidney and also showed a non-significant elevation (×6) in the heart (Figure 1). Total urinary UA was 34.3 ± 2.1 (range 31.8 to 35.8) μg UA per µmol creatinine in the wild type mice and 37.7 ± 11.1 (range 29.4 to 50.4) μg UA per µmol creatinine in the MPS IIIA mice.

### 2.2. Distribution of Di- to Hexasaccharides in Mouse Tissues and Urine

**Figure 2:**
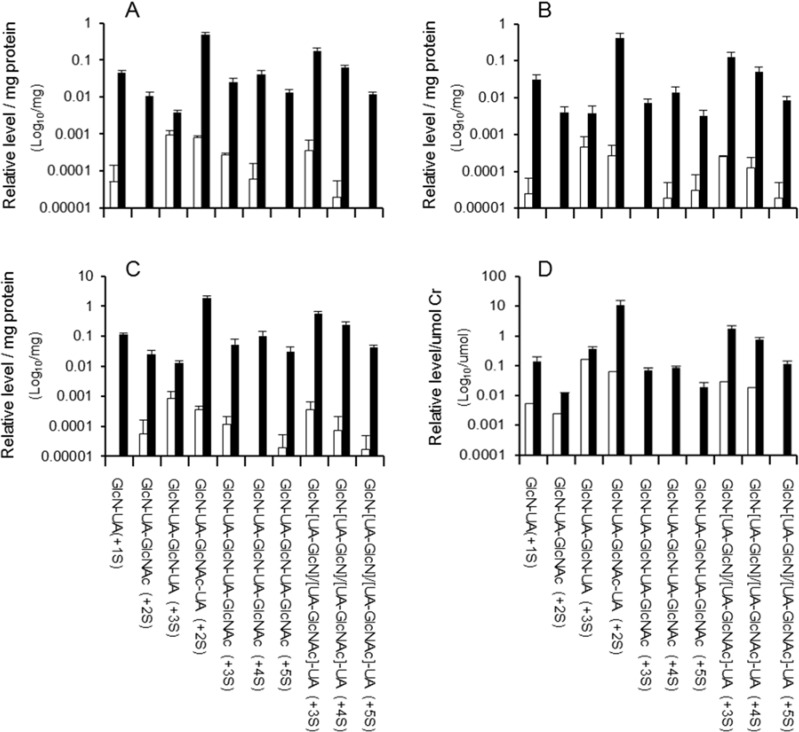
Relative oligosaccharide levels in wild type and MPS IIIA mice. Combined UA positive elutions from the anion exchange chromatography were analyzed for oligosaccharides by RP-HPLC-ESI-MS/MS as described under Materials and methods. Data points represent the relative levels of di- to hexasaccharides for wild type (open bars, *n* = 2 or 3) and three MPS IIIA mice (filled bars) in brain (panel (**A**)), lung (panel (**B**)), kidney (panel (**C**)), and urine (panel (**D**)). Oligosaccharide levels in some wild type tissues and urines were below the limit of detection (data not shown).

The relative abundance of each oligosaccharide in the MPS IIIA brain, lung and kidney was similar (Figure 2A–C), and similar trends were observed in the relative signal intensities in the spleen, heart and liver (data not shown). Relative urinary oligosaccharide levels in the MPS IIIA mouse showed a similar pattern to the tissues (compare Figure 2A–C with Figure 2D). Oligosaccharides with a peak area below 50 (or signal: noise of less than 5:1) were deemed below the detection limit of the analysis and were reported as not detected. Of the 10 oligosaccharides identified in the MPS IIIA mouse tissues and urine (Figure 2A–D) only five were detected in the wild type mouse heart, seven in brain, eight in lung and kidney, nine in spleen and liver, and six in urine.

**Figure 3:**
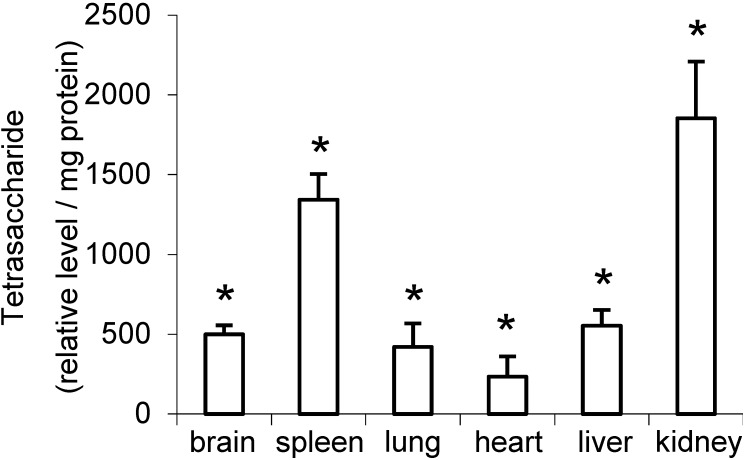
Relative level of tetrasaccharide (GlcN-UA-GlcNAc-UA (+2S)) in MPS IIIA mouse tissues. Combined UA positive elutions from the anion exchange chromatography were analyzed for oligosaccharides by RP-HPLC-ESI-MS/MS as described under Materials and methods. Data points represent the mean plus one standard deviation for MPS IIIA mice (*n* = 3). *p ≤ 0.05 compared to wild type mice.

The tetrasaccharide containing two sulfates (+2S) had the highest signal intensity in each MPS IIIA mouse tissue and was up to 210-fold higher than the tetrasaccharide (+3S), which had the lowest signal intensity. The relative levels of most di- to hexasaccharides were significantly elevated (*p* ≤ 0.05) in all MPS IIIA tissues and urine when compared to the corresponding wild type tissues. However, the tetrasaccharide (GlcN-UA-GlcN-UA (+3S)) was not significantly elevated in the lung (Figure 2B), heart or urine (Figure 2D) and the trisaccharide and pentasaccharides were not significantly elevated in the spleen. The relative levels of the di- to hexasaccharides varied up to 10-fold between the different tissues (Figure 3). To determine the relationship between the different oligosaccharides, Spearman’s correlation coefficients were calculated using the combined data from each tissue, which revealed a strong correlation between di-, tri-, tetra-, and hexasaccharides (0.83 to 0.97, *p* < 0.01) and a weaker correlation between di- and pentasaccharides (0.56, *p* < 0.05). A strong correlation was also observed between the tri- and pentasaccharides (0.86, *p* < 0.01).

Oligosaccharide levels in tissues could not be directly compared to urines as the relative levels are expressed differently (per mg protein in tissues and per µmol creatinine in urine). To permit comparison, levels of oligosaccharides in tissues and urines were expressed as a percentage of the total signal intensity of the di- to hexasaccharides. Table 1 shows the percentages of di-, tri-, tetra-, penta-, and hexasaccharides were comparable across the different tissues and urine tested. While the percentage of the disaccharide and pentasaccharides was lower in the urine compared to the average for all tissues examined, they were not statistically significant.

**Table 1:** Relative levels^a^ of di- to hexasaccharides in MPS IIIA mouse tissues and urine.

Structure of oligosaccharide ^b^	Brain ^c^	Spleen ^c^	Lung ^c^	Heart ^c^	Liver ^c^	Kidney ^c^	Average for all Tissues	Urine ^c^
GlcN-UA (+S)	5.0	4.8	4.6	5.2	6.0	3.7	5.0	2.1
GlcN-UA-GlcNAc (+2S)	1.1	0.7	0.6	0.6	0.9	0.8	0.8	0.9
GlcN-UA-GlcN-UA (+3S)	0.4	0.4	0.5	0.4	0.5	0.4	0.4	1.1
GlcN-UA-GlcNAc-UA (+2S)	56.1	75.2	62.7	58.2	65.6	61.3	63.2	64.4
GlcN-UA-GlcN-UA-GlcNAc (+3 to+5S)	8.9	2.5	3.7	5.2	4.2	6.1	5.0	3.4
GlcN-[UA-GlcN]/[UA-GlcNAc]-UA (+3 to +5S)	28.5	16.4	27.9	30.4	22.8	27.7	25.6	28.0

^a^: Relative levels calculated from the signal intensities for each oligosaccharide assuming equal response factors and expressed as a percentage of the total signal intensity; ^b^: GlcN, glucosamine; UA, uronic acid; GlcNAc, N-acetyl glucosamine; (+nS), refers to the number of sulfate (SO_3_) groups in the oligosaccharide structure; ^c^ Results expressed as a percentage of total oligosaccharides measured (average of *n* = 3).

### 2.3. Correlation between HS Oligosaccharides and UA in MPS IIIA Mouse Tissues

**Figure 4:**
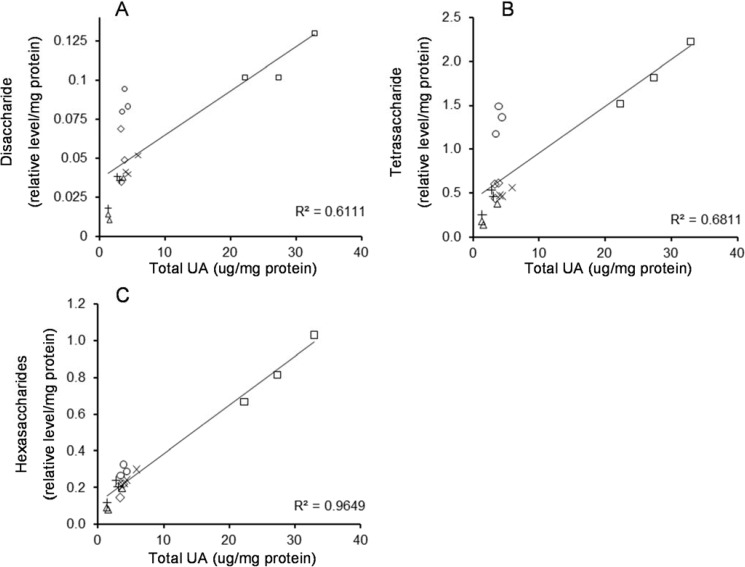
Relationship between sulfated oligosaccharides and total UA for MPS IIIA mouse tissues. Relative levels of disaccharide, tetrasaccharide (GlcN-UA-GlcNAc-UA (+2S)) and combined hexasaccharides (+3 to +5S) were plotted against total UA (UA equivalents) for the MPS IIIA tissues. The relationship between the disaccharide (panel (**A**)), tetrasaccharide (panel (**B**)) and hexasaccharides (panel (**C**)) and UA for the kidney (□), brain (x), liver (◊), lung (+), spleen (○) and heart (∆) are shown.

To investigate the relationship between the relative levels of oligosaccharides and total UA in the tissues, the relative levels of di-, tetra-, and hexasaccharides were plotted against total UA (Figure 4). On the basis of strong Spearman coefficients (0.91 to 0.93, *p* < 0.01), the relative levels of the hexasaccharides 3, 4, and 5S were combined to give a result for total hexasaccharides. A weak relationship was observed between the disaccharide and UA (R^2^ 0.61), with kidney and spleen clearly separated from brain, liver, lung and kidney (Figure 4A). The tetrasaccharide and UA also showed a weak linear correlation (R^2^ 0.68) with the same two tissues separated from the other four tissues (Figure 4B), while the hexasaccharides and UA showed the strongest linear relationship (R^2^ 0.97, Figure 4C).

## 3. Experimental Section

### 3.1. Materials

CHCl_3_ (HPLC grade), LiCl and CH_3_COONH_4_ (analytical grade) were supplied by Ajax FineChem (Seven Hills, NSW, Australia). 1-phenyl-3-methyl-5-pyrazolone (PMP) was purchased from Tokyo Kasei Kogyo (Tokyo, Japan). DEAE Sephacel resin and the internal standard 4-deoxy-L-threo-hex-4-enopyranosyluronic(1→3)N-acetyl-galactosamine-4-sulphate (∆UA-GalNAc (4S)) were purchased from Sigma-Aldrich (St. Louis, MO, USA).

### 3.2. Animals

MPS IIIA and wild type mouse tissues and urine were obtained from the archival tissue bank taken from mice that were excess to the breeding colony at the Women’s and Children’s Hospital in Adelaide. MPS IIIA and wild type mouse tissues (kidney, liver, brain, heart, lung, spleen) and urine werestored at −70 °C. All animals were handled/housed in accordance with the Ethical Guidelines of the National Health and Medical Research Council of Australia and with consent of the institutional AnimalEthics Committee.

### 3.3. Isolation of Oligosaccharides from Tissues and Urine

Tissues from MPS IIIA (*n* = 3) and wild type mice (*n* = 3), aged 30 weeks and urine from MPS IIIA (*n* = 3) and wild type mice (*n* = 2), aged 24 to 30 weeks were stored at −20 °C. Whole tissues (69 to 672 mg) were homogenized in 1.5 mL of 0.25 M LiCl, subjected to six freeze/thaw cycles and centrifuged at 13,000 g for 10 min. Total protein was determined using a BCA protein assay kit (Progen Biosciences, Archerfield, QLD, Australia). Urinary creatinine was measured on a Beckman Synchron CX5 Chemistry Analyzer (Beckman Coulter, Brea, CA, USA). Mouse urine samples (400 to 600 μL) and tissue supernatants were diluted to 5 mL with acetate buffer (100 mM CH_3_COONH_4_, pH 5) and mixed with 1 mL of DEAE Sephacel (previously equilibrated with acetate buffer) at 4 °C on a rotator overnight. The slurry was poured into Poly Prep chromatography columns (0.8 × 4 cm OK, Bio-Rad, Hercules, CA, USA), and the columns washed with acetate buffer (5 mL). Oligosaccharides were eluted with 1.2 M LiCl in acetate buffer (9 × 0.5 mL) followed by 2 M LiCl in acetate buffer (1 × 3 mL).

### 3.4. Total UA in Tissues and Urine

Total urinary and tissue GAG was determined using the hydroxydiphenyl method to measure free and conjugated UA [2]. All fractions from the DEAE Sephacel eluate were assayed and the UA containing fractions (2–5) were combined for further analysis.

### 3.5. Derivatization of Oligosaccharides

Aliquots of the UA containing DEAE Sephacel eluate (500 μL) were lyophilized and oligosaccharides were derivatized with PMP, containing 2 nmol of ∆UA-GalNAc (4S) as internal standard, as previously described [7] with minor modification. Derivatized oligosaccharides were not subjected to solid phase extraction. After removal of unincorporated PMP, 400 µL of the upper aqueous layer was removed, lyophilized and stored at −20 °C prior to HPLC ESI-MS/MS analysis.

### 3.6. HPLC ESI-MS/MS Analysis of di- to Hexasaccharides

Reverse-phase HPLC analysis of the derivatized oligosaccharides was performed as previously described [20] with the following modifications. Derivatized oligosaccharides were resuspended in 100 μL of H_2_O, centrifuged (13,000 g, 1 min) and injected (20 µL) into a stream of 0% mobile phase B and loaded onto the HPLC column. After de-salting the samples for 5 minutes at 0% B, a linear elution gradient of up to 100% B was established between 5.1 and 10 min and then the column was re-equilibrated with 0% B from 10.1 to 15 min. Mobile phases were delivered at 0.2 mL/min using Agilent series 1100 pumps. The elution profiles of the PMP-oligosaccharides in the HPLC separations were monitored by ESI-MS/MS in the negative ion multiple reaction-monitoring (MRM) mode on a PE Sciex API 3000 triple-quadrupole mass spectrometer equipped with Analyst software (Version 1.3) and a turbo-ionspray source. Nitrogen was used as the auxillary, curtain and collision gas. The ion source temperature was set to 200 °C and the ion spray voltage was set to −4500 V. Each MRM pair was monitored for 100 ms at unit resolution. Relative oligosaccharide levels were determined by relating the peak areas of the PMP-oligosaccharides to the peak area of the PMP-∆UA-GalNAc4S internal standard (multiplying by a factor of 1000) and expressed as relative units per mg protein for the mouse tissues and relative units per µmol creatinine for urine. The 10 oligosaccharides measured were previously identified in the urine of an MPS IIIA patient and collision activated dissociation was performed on each oligosaccharide to enable partial structural characterization and to identify a product ion pair (MRM pair) for quantitation [20].

### 3.7. Statistical Analysis

All statistical analyses were performed using the Independent-samples *T*-test, Spearman correlation coefficient or linear regression. Results were considered significant at *p* < 0.05. Analyses were performed using SPSS (Version 19 for Windows) statistical software (IBM Inc., Chicago, IL, USA, Country).

## 4. Discussion

Anion exchange chromatography enabled separation of oligosaccharides from proteins and other compounds in the tissue homogenates and urine resulting in enriched oligosaccharide fractions. The inclusion of RP-HPLC enabled the removal of salts prior to mass spectrometry, which minimized signal suppression and led to increased sensitivity as evidenced by the increase in the range of the relative level of disaccharide (normalized to protein) detected from 10 to 100 compared to the earlier reported range of one and two [14]. In previous studies, the removal of salts from derivatized oligosaccharides by solid phase extraction favored the recovery of mono- and disaccharides [7,14] with loss of larger oligosaccharides from the solid phase. The use of RP-HPLC to remove the salts minimized the loss of the larger oligosaccharides. The tetrasaccharide (GlcN-UA-GlcNAc-UA (+2S)) was shown to give the highest signal in all tissues demonstrating that this oligosaccharide provides the greatest analytical sensitivity of the di- to hexasaccharides measured here using RP-HPLC ESI-MS/MS. The relative levels of oligosaccharides were reported due to the non-availability of appropriate stable isotope internal standards.

We used 30-week-old mice with advanced MPS IIIA disease and demonstrated that the relative level of the disaccharide was highest in the kidney, with decreasing levels in spleen, liver, brain and lung with the lowest level in heart. The distribution of oligosaccharides, when ranked from highest to lowest by signal intensity was similar across the different tissues (Figure 2 and Figure 3), which suggests that the endo- and exo-enzymes responsible for the production of the oligosaccharides are similar in the tissues. However, the relative level of each oligosaccharide varied up to 10-fold between tissues, with the kidney containing the highest levels and heart containing the lowest. Although MPS IIIA patients do not manifest kidney disease, the high levels of tetrasaccharides observed in the kidney may reflect the collective sum of tetrasaccharides eliminated from the tissues throughout the body. HS-derived oligosaccharides are more likely to reflect true substrate load within the different tissues as opposed to the measurement of total UA, which includes the measurement of all GAG species including chondroitin sulfate, dermatan sulfate and HS. Nonetheless, this was supported by the correlation with the total UA (Figure 4) and was consistent with the elevated HS derived oligosaccharides in these tissues and with the GAG levels previously reported in the MPS IIIA mouse [5].

The di- and tetrasaccharide/total UA ratios varied between tissues whereas the hexasaccharide/total UA ratio was more consistent. This implies that the hexasaccharide may more accurately reflect total UA possibly due to the influence of HS structure on the generation of different oligosaccharides via the action of endo-enzymes. Longer oligosaccharides might reflect less modification by other enzymes and so their compositions may be closer to that of the GAG. The different oligosaccharide/total UA ratios observed in spleen appear to relate to the elevation in oligosaccharide in this tissue rather than decreased total UA: it may also relate to endo-enzyme activity that may be elevated in spleen relative to other tissues as a result of the high GAG content. Several endo-ß-glucuronidases, known as heparanases, have been shown to degrade HS [21,22,23,24,25]. Heparanase cleaves at glucuronosyl bonds within HS resulting in smaller saccharide chains and specificity towards different areas of sulfation within the HS chain. The resulting partially degraded oligosaccharides are then subjected to exo-enzyme digestion to produce monosaccharides and sulfate. Heparanase upregulation has been reported in cancer [26,27], diabetic nephropathy [28], and inflammation [29,30]. Pathways, such as inflammation or oxidative stress, have been highlighted as a major component of the neuropathology observed in the MPS IIIA mouse [31]. Quantification of heparanase in tissue extracts and urine from the MPS IIIA mouse may therefore be of experimental and clinical significance.

In urine, we observed similar levels of total UA in wild type and MPS IIIA mice which contrasts with the findings of Bhaumik *et al*. [32] who demonstrated increased HS in MPS IIIA mice by high resolution gel electrophoresis. The chondroitin sulfate and dermatan sulfate present in the urine also contributed to the total UA measured in our study, which may be masking the effect of the increase in HS for MPS IIIA. Roberts *et al*. [3] also reported elevations in urinary GAG in MPS IIIA mice using cetylpyrinium chloride and ethanol precipitation. However, this approach is likely to include only HS oligosaccharides larger than hexasaccharides and GAG whereas the anion exchange used in our study would isolate all mono and oligosaccharides in urine [20]. In support of our finding in MPS IIIA urine, Li *et al*. [33] also reported similar levels of GAG in the urine of wild type and MPS IIIB mice.

Comparison of the relative level of oligosaccharides in tissues and urine of the MPS IIIA mouse showed that the di- to hexasaccharides were similar in the brain, lung, heart, spleen, liver, kidney, and urine. As all tissues have similar profiles of oligosaccharides we cannot ascribe the urine oligosaccharides to any of the specific tissues studied. Further investigation is required to resolve this question.
